# Characterization of a New Multifunctional GH20 β-*N*-Acetylglucosaminidase From *Chitinibacter* sp. GC72 and Its Application in Converting Chitin Into *N-*Acetyl Glucosamine

**DOI:** 10.3389/fmicb.2022.874908

**Published:** 2022-05-10

**Authors:** Yan Chen, Ning Zhou, Xueman Chen, Guoguang Wei, Alei Zhang, Kequan Chen, Pingkai Ouyang

**Affiliations:** ^1^State Key Laboratory of Materials-Oriented Chemical Engineering, College of Biotechnology and Pharmaceutical Engineering, Nanjing Tech University, Nanjing, China; ^2^Jiangsu Key Laboratory of Marine Bioresources and Environment, Jiangsu Ocean University, Lianyungang, China

**Keywords:** *N*-acetyl glucosamine, β-*N*-acetylglucosaminidase, transglycosylation activity, chitin, biochemical characterization, synergistic action

## Abstract

In this study, a gene encoding β-*N*-acetylglucosaminidase, designated NAGaseA, was cloned from *Chitinibacter* sp. GC72 and subsequently functional expressed in *Escherichia coli* BL21 (DE3). NAGaseA contains a glycoside hydrolase family 20 catalytic domain that shows low identity with the corresponding domain of the well-characterized NAGases. The recombinant NAGaseA had a molecular mass of 92 kDa. Biochemical characterization of the purified NAGaseA revealed that the optimal reaction condition was at 40°C and pH 6.5, and exhibited great pH stability in the range of pH 6.5–9.5. The *V*_*ma*_*_*x*_*, *K*_m_, *k*_cat_, and *k*_cat_*/K*_m_ of NAGaseA toward *p*-nitrophenyl-*N*-acetyl glucosaminide (*p*NP-GlcNAc) were 3333.33 μmol min^–1^ l^–1^, 39.99 μmol l^–1^, 4667.07 s^–1^, and 116.71 ml μmol^–1^ s^–1^, respectively. Analysis of the hydrolysis products of *N*-acetyl chitin oligosaccharides (*N*-Acetyl COSs) indicated that NAGaseA was capable of converting *N*-acetyl COSs ((GlcNAc)_2_–(GlcNAc)_6_) into GlcNAc with hydrolysis ability order: (GlcNAc)_2_ > (GlcNAc)_3_ > (GlcNAc)_4_ > (GlcNAc)_5_ > (GlcNAc)_6_. Moreover, NAGaseA could generate (GlcNAc)_3_–(GlcNAc)_6_ from (GlcNAc)_2_–(GlcNAc)_5_, respectively. These results showed that NAGaseA is a multifunctional NAGase with transglycosylation activity. In addition, significantly synergistic action was observed between NAGaseA and other sources of chitinases during hydrolysis of colloid chitin. Finally, 0.759, 0.481, and 0.986 g/l of GlcNAc with a purity of 96% were obtained using three different chitinase combinations, which were 1.61-, 2.36-, and 2.69-fold that of the GlcNAc production using the single chitinase. This observation indicated that NAGaseA could be a potential candidate enzyme in commercial GlcNAc production.

## Introduction

Chitin is the second most abundant polysaccharide on earth after cellulose, it is mainly derived from fungal cell walls, insect exoskeletons, and the crab and shrimp shells. An estimated 10^10^–10^11^ tons of chitin are produced per year (Anitha et al., 2014). However, 35–45% of chitin biomass is discarded as waste due to a lack of efficient refinery protocols, which leads to waste of resources and severe environmental problems (Wei et al., 2017; Zhou et al., 2017a). *N*-acetyl glucosamine (GlcNAc), the monomeric unit of chitin, possesses many specific bioactivities and has been widely used in biomedical, food, and chemical industries (Bhattacharya et al., 2007; Suresh and Kumar, 2012; Kisiel and Kepczynska, 2017). Therefore, it is of economic and environmental value to realize the efficient production of GlcNAc from abundant chitin resources (Gao et al., 2018).

Commercial GlcNAc was often produced *via* acid hydrolysis of chitin. However, this protocol is difficult to directly obtain GlcNAc owing to the deacetylation of the *N*-acetyl group of products (Aam et al., 2010). In this case, chitin is first hydrolyzed to GlcN, and then chemical acetylated to form GlcNAc. This multistep process not only results in low yield, high cost, and poor biological activity of products but also leads to numerous environmental issues (Chen et al., 2010; Kim et al., 2017). Alternatively, enzymatic hydrolysis of chitin into GlcNAc using chitinolytic enzymes was shown to be a more attractive approach in recent years, because of the green process and the excellent bioactivity of the product (Park et al., 2011).

Chitinolytic enzymes are complex enzyme systems with a good synergistic effect, which could be classified into three types: endo-acting chitinases that cut randomly chitin chains to generate *N*-acetyl chitin oligosaccharides (*N*-acetyl COSs); progressive exo-acting chitinases that release GlcNAc dimer from the non-reducing or reducing end of chitin chains; NAGases that hydrolyze *N*-acetyl COSs or GlcNAc dimer into GlcNAc (Zhou et al., 2017a; Lv et al., 2019; Liu et al., 2020). Among, NAGase plays a key role in the control of the ratio and yield of GlcNAc during the hydrolysis process of chitin. Thus, it is of great significance to excavate NAGase with high activity for efficiently converting *N*-acetyl COSs into GlcNAc.

In our previous study, chitinolytic enzymes were derived from the bacterium *Chitinibacter* sp. GC72 isolated from pond mud in Nanjing were capable of hydrolyzing chitin into GlcNAc as the sole product (Gao et al., 2015). Moreover, only one gene encoding NAGase, named NAGaseA, was found in strain GC72 *via* complete genome sequencing and analysis (Zhang et al., 2020a). In this study, the NAGaseA gene was cloned from the genome of strain GC72 and heterologously expressed in *Escherichia coli* (BL21). The enzymatic properties and hydrolysis mode of the recombinant NAGaseA were investigated. Furthermore, the synergetic effect between NAGaseA and various chitinases in converting chitin to produce GlcNAc was also studied. This study provided a possible application in the enzymatic production of GlcNAc.

## Materials and Methods

### Chemicals, Strains, and Plasmids

Chitin powder, 4-methylumbelliferyl *N*-acetyl glucosaminide (4-MU-GlcNAc), and *p*NP-acetyl galactosaminide (*p*NP-GlcNAc) were purchased from Aladdin Reagent Co., Ltd. (Shanghai, China). *N*-acetyl chitooligosaccharides (*N*-acetyl-COSs) standards ranging from dimer to hexamer were purchased from Qingdao BZ Oligo Biotech Co., Ltd. (Qingdao, China). The molecular reagents were purchased from Takara Bio Inc. (Dalian, China). All chemicals used in this study were of analytical grade or higher purity. Colloidal chitin was prepared from chitin powder according to the methods described by Gao et al. (2015).

*Escherichia coli* Trans1-T1, BL21 (DE3), and the expression vector pET-28a (+) plasmid were purchased from Novagen. The strain *Chitinibacter* sp. GC72 (CCTCC M 2014113) and *Chitinolyticbacter meiyuanensis* SYBC-H1 (ATCC BAA-2140) used in this study were isolated and stored in our laboratory (Hao et al., 2011; Gao et al., 2015).

### Culture Conditions

Strains GC72 and SYBC-H1 were cultured according to our previous study (Hao et al., 2011; Gao et al., 2015). *E. coli* strains were routinely cultivated aerobically at 37°C in LB medium (10 g/l tryptone, 5 g/l yeast extract, and 5 g/l NaCl) or agar plates containing 50 μg/ml kanamycin.

### Molecular Cloning and Sequence Analysis

The genomic DNA of strain GC72 was extracted using a bacteria genome extraction Kit (TIANGEN, China) and was used as the PCR template. Two primers used to amplify the NAGaseA were synthesized by Genscript Biotech (Nanjing, China) and the sequences were as followed: forward primer 5′-GTGCCGCGCGGCAGCCATATGAACAAGCCAGCAGGT-3′; reserve primer 5′-GTGGTGGTGGTGCTCGAGCACCGCAAC CACCCGGCT-3′. The PCR system and conditions were as follows: 94°C for 5 min, followed by 30 cycles of 95°C for 30 s, 55°C for 30 s, and 72°C for 1 min, and a final extension at 72°C for 10 min. The NAGaseA gene generated from PCR and the plasmid pET-28a (+) was double digested with *Nde*I and *Xho*I, followed by a ligation using the ClonExpressTM II/One Step Cloning Kit (Vazyme, China). The recombinant plasmid was transformed into *E. coli* Trans-T1 competent cells and sequenced by Genscript Biotech (Nanjing, China).

Nucleotide and amino acid sequences were analyzed using Snap Gene™ 1.1.3 software^1^ and the ExPASy protparam tool.^2^ The DNA and protein sequence alignments were performed *via* the NCBI server with the programs BLASTN and BLASTP,^3^ respectively. Phylogenetic trees were inferred using the neighbor-joining algorithm in MEGA 7. The conserved domains and the GH family classification were identified *via* the website.^4^ The signal peptide was predicted in the SignalP 4.1 server.^5^ Protein homologous sequences alignment was carried out using ClustalX 2.1 software and ESPript 3.0.^6^ The structure of NAGaseA was predicted with RaptorX.^7^

### Expression and Purification of Recombinant NAGaesA

The recombinant plasmid pET-28a (+) harboring NAGaesA gene was transformed into *E. coli* BL21(DE3), incubated in LB liquid medium (containing 50 μg/ml kanamycin), and then cultured at 37°C with shaking at 200 rpm. When the optical density (OD_600_) of the culture medium was approximately 0.6, isopropyl β-D-thiogalactoside was added to a final concentration of 1 mM for protein induction, and the culture was incubated overnight at 18°C with shaking at 200 rpm.

Cultures were harvested by centrifugation at 6,000 × *g* and 4°C for 10 min, after which the pellet was gently resuspended in binding buffer A (50 mM phosphate buffer, 500 mM NaCl, 50 mM imidazole, pH 7.4) and lysed by JY92-IIN ultrasonication (Ningbo Xinzhi Biotechnology, Ltd., Ningbo, China). The cell debris was removed by centrifugation at 12,000 × *g* for 10 min at 4°C and the supernatant was retained as a crude enzyme. The recombinant NAGaseA was purified using a fast protein liquid chromatography 448 system (GE AKTA Pure 150; General Electric Co., IA, America with a Ni-449 nitrilotriacetic acid affinity chromatography (Ni-NTA) column (His Trap™ FF 5 ml). The supernatant was filtered with a 0.22 μm membrane before being loaded onto a Ni Sepharose column. The NAGaseA protein was eluted with buffer B (50 mM sodium phosphate, 500 mM NaCl, 500 mM imidazole, and pH 7.4) under a flow rate of 3 ml/min. The eluted protein was collected, concentrated, and exchanged with 20 mM phosphate buffer (pH 7.0) *via* ultrafiltration and stored at 4°C before use (Zhang et al., 2020b).

Sodium dodecyl sulfate-polyacrylamide gel electrophoresis (SDS-PAGE) was performed to identify the target protein, and the protein concentration was determined using the Bradford method (Bradford, 1976).

### Enzyme Assay and Substrate Specificity of Recombinant NAGaesA

NAGaseA activity was assayed using *p*NP-GlcNAc as substrate. A total of 1 ml reaction mixture containing 50 μl *p*NP-GlcNAc (10 mM) and 10 μl of purified NAGaseA in 50 mM phosphate-buffered saline (PBS) buffer (pH 7.0). The mixture was incubated at 40°C for 10 min and then 1 ml NaOH (1 M) solution was added to terminate the reaction. The amount of *p*NP released was determined under the absorbance measured at 405 nm according to our previous reported (Zhang et al., 2018). One unit of NAGase activity was defined as the amount of enzyme required to release 1 μmol *p*NP per minute under the assay conditions.

Chitinase activity was measured using colloid chitin as the substrate. The 1 ml reaction mixture was performed with 0.2 ml of enzyme and 0.3 ml colloid chitin (10 g/l) in 50 mM phosphate buffer (pH 7.0). The reaction was conducted at 37°C for 30 min, and then 1 ml of 3,5-dinitrosalicylic acid (DNS) was added to the mixture followed by boiling at 100°C for 5 min (Breuil and Saddler, 1985).

### Enzymatic Characterization of Recombinant NAGaseA

The enzymatic characterization of recombinant NAGaseA was performed using *p*NP-GlcNAc as the substrate. To determine the optimum temperature of NAGaseA, the reaction was incubated under interval temperatures ranging from 30°C to 80°C in 50 mM PBS (pH 6.5). The thermostability of NAGaseA was determined by measuring the residual activity at pH 6.5 and 40°C after the enzyme was treated in 50 mM sodium citrate (pH 6.5) for 12 h at different temperatures.

The optimal pH of NAGaseA was determined at 40°C under a pH range of 3.0-11.0 using different buffers (50 mM): citrate buffer (pH 3.0-6.0), phosphate buffer (pH 5.5-8.0), Tris-HCl buffer (pH 7.0-9.0), and glycine-NaOH buffer (pH 8.5-11.0). To determine the pH stability, the enzyme was incubated with various pH buffers, and the residual activities were measured by the standard assay [citrate buffer (pH 6.0); phosphate buffer (pH 6.5); phosphate buffer (pH 7.0); phosphate buffer (pH 8); glycine-NaOH buffer (pH 9); glycine-NaOH buffer (pH 10)].

To determine the effect of different metal ions on NAGaseA activity, the purified enzyme was incubated with 10 mM EDTA at 40°C for 30 min, and then remove the EDTA using 50 mM PBS (pH 7.0) by ultrafiltration. After that, the metal-free NAGaseA was incubated with various metal salts containing Ca^2+^ (CaCl_2_), Co^2+^ (CoCl_2_), Mn^2+^ (MnCl_2_), Cu^2+^ (CuCl_2_), Fe^3+^ (FeCl_3_), Mg^2+^ (MgCl_2_), Zn^2+^ (ZnCl_2_), Al^3+^ (AlCl_3_⋅6H_2_O), and Ni^2+^ (NiCl_2_) at a final concentration of 10 mM for 30 min. The residual activities were measured using *p*NP-GlcNAc at 40°C in 50 mM PBS (pH 6.5) for 30 min, and the residual activity of NAGaseA without metal ions incubation was used as the control (100%).

The kinetics parameters were determined by measuring the enzyme activity toward *p*NP-GlcNAc at 40°C in 50 mM PBS (pH 6.5) for 10 min using different concentrations of substrate (50-2,500 μM) as the substrate. The values of *V*_max_, *K*_m_, and *k*_cat_ were estimated by linear regression from double-reciprocal plots according to the method of Lineweaver (Price, 1985).

### Hydrolytic Pattern of Recombinant NAGaseA

The reaction mixtures containing purified NAGaseA (60 ng) and various substrates ((GlcNAc)_2_-(GlcNAc)_5_) at a final concentration of 10 g/l were incubated at 40°C at various time intervals. In each case, the supernatant after hydrolysis was diluted with 50% acetonitrile and centrifuged at 8,000 × *g* for 10 min to remove the protein. The hydrolysis products were analyzed by Agilent 1260 series HPLC system according to our previous study (Zhang et al., 2018).

### Cooperative Interaction Analysis of NAGaseA With Other Chitinases

The fermentation broth of strain GC72 and SYBC-H1 was centrifuged at 12,000 × *g* for 15 min at 4°C, and the supernatant was collected as a crude enzyme before use. Exochitinase ChiA from *Serratia proteamaculans* (stored in our laboratory) was cloned, expressed, and purified as previously reported (Purushotham et al., 2012).

The cooperative interaction between NAGaseA and other sources of chitinases derived from strain *Serratia proteamaculans* (recombinant ChiA), strain SYBC (crude enzyme), and strain GC72 (crude enzyme) were determined using colloid chitin as the substrate. The reaction mixture (1 ml) contained colloidal chitin with a final concentration of 10 g/l and either 50 μl NAGaseA (4.8 U/ml reaction system), 50 μl ChiA (6.1 U/ml reaction system), 50 μl SYBC chitinase (2.8 U/ml reaction system), and 50 μl GC72 chitinase (5.2 U/ml reaction system) or both enzymes and was incubated at 40°C in 50 mM PBS (pH 6.5) for 30 min. The amount of reducing sugars released was measured using the DNS method and HPLC mentioned above.

The GlcNAc purity was calculated as the following formula: GlcNAc purity (%) = GlcNAc (g)/(GlcNAc)_1–2_ released (g) × 100.

## Results and Discussion

### Cloning of the NAGaseA Gene and Sequence Analysis

Based on the gene function prediction of the complete genome of *Chitinbacter* sp. GC72, ORF 159 was annotated as a potential β-*N*-acetylglucosaminidase (NAGaseA) gene. The total length of NAGaseA gene is 2, 535 bp, encoding 844 amino acids. After PCR, the same nucleic acid sequence was obtained, which indicated that NAGaseA gene was successfully cloned. Besides, the predicted molecular weight and theoretical pI of NAGaseA were 92.4 kDa and 5.24, respectively.

According to the result of BLASTP analysis of the amino acid sequence, NAGaseA belonged to glycoside hydrolase (GH) family 20 (GH20) and shared the highest identity of 94.43% with the putative GH20 NAGase from *Chitinibacter fontanus* (WP_180317904), followed by 88.27% with GH20 NAGase from *Chitinibacter* sp. ZOR0017 (WP_047394852). However, the relative enzymatic characterization of these proteins has not been reported. Among the studied NAGases, NAGaseA displayed the highest identity (68.84%) with GH20 NAGase from *Aeromonas sp.* 10S-24 (accession no. BAA92145; Ueda et al., 2000), followed by 67.65% with GH20 NAGase from *C. meiyuanensis* (accession no. WP_148716590; Zhang et al., 2018), 32.83% with GH20 NAGase from *Serratia marcescens* (PDB 1QBA; Tews et al., 1996b), 30.79% with GH20 NAGase from *Vibrio harveyi* (PDB 6EZR; Suginta et al., 2010), and 27.05% with GH20 NAGase from *Microbacterium sp*. HJ5 (PDB 7BWG; Zhou et al., 2017b). A phylogenetic tree of NAGaseA with some putative and verified GH20 family NAGases was further constructed based on their sequence similarities. The results suggested that NAGaseA shared low sequence similarity with most experimentally characterized GH20 NAGases (Supplementary Figure 1).

The result of multiple alignments of NAGaseA with other GH20 NAGases was shown in Supplementary Figure 2. The typical acidic pairs D512-E513 in NAGaseA are completely aligned with many other functionally characterized GH20 NAGases, which probably functioned as the catalytic residues. In addition, other highly conserved residues among NAGase species, namely, R319, H426, V467, Q468, W562, W600, Y625, D627, L628, Y639, W641, W698, and E700 were also observed, which may play an important role in binding the GlcNAc ligand (Prag et al., 2000). Furthermore, the consensus H-X-G-G motif before the catalytic residue in NAGaseA is highly conserved among the catalytic domain of GH20 NAGases. Based on the analysis of secondary structure, NAGaseA possesses 20 α-helices and 30 β-sheets with the typical (β/α)_8_ barrel fold in the GH20 catalytic domain, which is highly consistent with various GH20 NAGases from different sources (Zhang et al., 2018).

**FIGURE 1:**
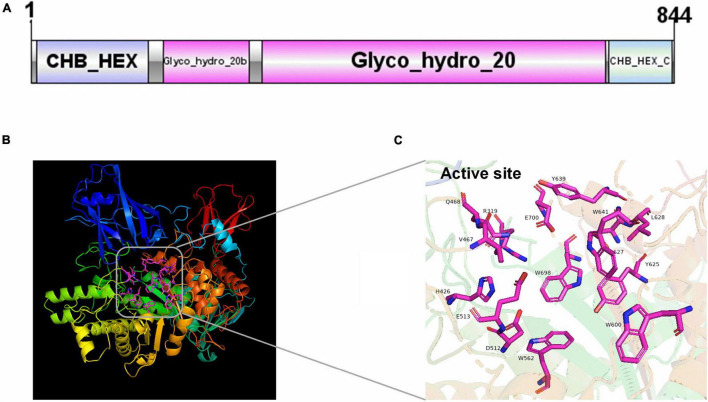
The domain and structure prediction of NAGaseA. **(A)** The conserved domain of NAGaseA, the image was generated using IBS Illustrator (Liu et al., 2015). **(B)** The prediction of the 3D structure of NAGaseA, **(C)** the active sites of NAGaseA.

The structure feature of NAGaseA was shown in Figure 1A. The predicted protein structure consisted of four domains as follows: domain I (CHB_HEX domain of residues 2–153); domain II (Glyco_hydro_20b domain of residues 174-287); domain III (Glyco_hydro_20 domain residues 308-726, the catalytic domain containing a TIM barrel fold); and domain IV (CHB_HEX_C domain of residues 758-841). As presented in Figure 1B, the model of NAGaseA was predicted based on the crystal structure of GH20 NAGase from *S. marcescens* (PDB 1QBA) with a protein identity of 34.22% (Tews et al., 1996b). The active pocket formed by R319, H426, V467, Q468, D512, E513, W562, W600, Y625, D627, L628, Y639, W641, W698, and E700 were labeled in the 3D structure model of NAGaseA (Figure 1C).

### Expression of NAGaseA Gene and Purification of Recombinant NAGaseA

**FIGURE 2:**
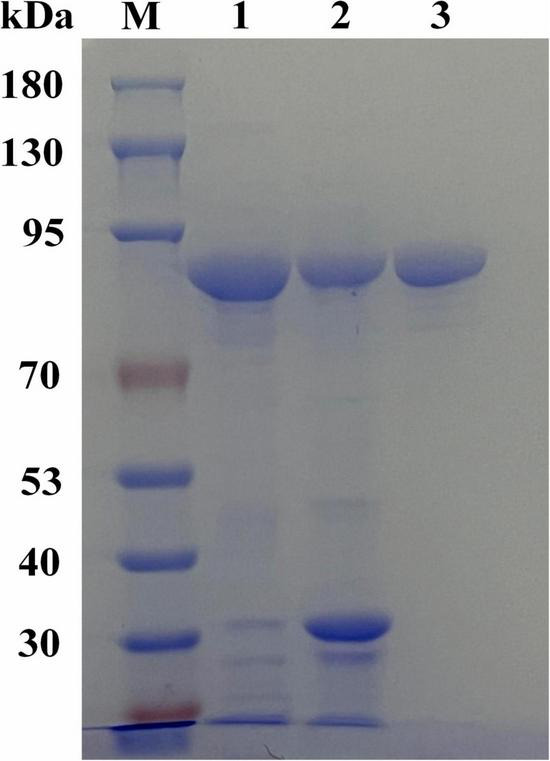
SDS-PAGE analysis of the expression and purification of recombinant NAGaseA. The amount of the loaded protein is 10 μg. Lane M, protein molecular mass marker, lane 1, crude enzyme preparation of NAGaseA, lane 2, crude enzyme deposit of NAGaseA, lane 3, NAGaseA purified by His6-tag affinity chromatography.

The gene encoding NAGaseA was successfully expressed as a soluble protein in *E. coli* BL21 (DE3). The SDS-PAGE analysis (Figure 2) showed that a single target protein band was obtained with a molecular weight of ∼92 kDa after Ni-NTA resin affinity purification, which was consistent with the 92,379 Da calculated from the amino acid sequence containing the His6-tag. This is different from that of some GH20 NAGases from *Microbacterium* sp. HJ5 (55.9 kDa; Zhou et al., 2017b), *Paenibacillus* sp. (57.5 kDa), *V. harveyi* 650 (73 kDa; Suginta et al., 2010), and *Streptomyces thermoviolaceus* (60 kDa; Kubota et al., 2004). However, the M_w_ of NAGaseA is similar to the previously reported GH20 NAGase from *C. meiyuanensis* with a molecular mass of 92,571 Da (Zhang et al., 2020b). The specific activity of recombinant NAGaseA exhibited a 1.39-fold increase from 270.17 to 373.29 U/mg with a protein recovery of 78.6% yield after purification (Supplementary Table 1).

### Effects of Temperature and pH on the Enzymatic Activity and Stability of Purified Recombinant NAGaseA

**FIGURE 3:**
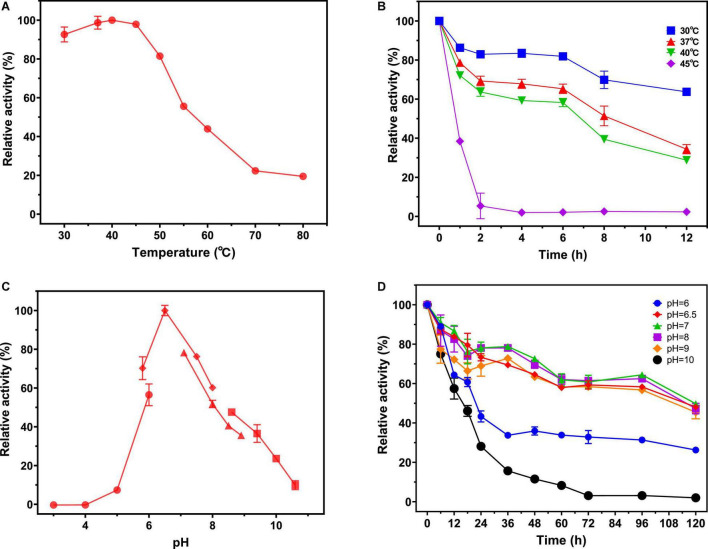
Effect of pH and temperature on the activity and stability of NAGaseA. **(A)** The optimal temperature of the recombinant NAGaseA. The temperature optimum was determined at different temperatures (30–80°C) in 50 mM phosphate buffer (pH 6.5). **(B)** The temperature stability of the recombinant NAGaseA. To determine the temperature stability, the enzyme was incubated in 50 mM sodium citrate (pH 6.5) for 12 h at different temperatures, the residual activity was measured at pH 6.5 and 40°C. **(C)** The optimal pH of the recombinant NAGaseA. The optimal pH was determined in 50 mM solutions of various buffers within the pH range 3–11. (⚫ Citrate buffer (pH 3.0–6.0), ◆Phosphate buffer (pH (6.0–8.0), ▲ Tris-HCl buffer (pH 7.0–9.0), ◼ Glycine-NaOH buffer (pH 8.5–10.5)). **(D)** The pH stability of the recombinant NAGaseA. To determine the pH stability, the enzyme was incubated with various pH buffers, and the residual activities were measured (⚫ Citrate buffer (pH 6.0), ◆Phosphate buffer (pH 6.5), ▲ Phosphate buffer (pH 7.0), ◼ Phosphate buffer (pH 8),◆Glycine-NaOH buffer (pH 9), ⚫Glycine-NaOH buffer (pH 10).

The temperature and pH profiles of recombinant NAGaseA were investigated in Figure 3. As shown in Figure 3A, the recombinant NAGaseA displayed the optimal temperature at 40°C, which was consisted of NAGase from *C. Meiyuanensis* SYBC-H1 (40°C; Zhang et al., 2020b), but different from NAGases from *S. marcescens* (52°C; Tews et al., 1996b), *Microbacterium* sp. HJ5 (45°C; Zhou et al., 2017b), *Streptomyces* sp. F-3 (60°C; Sun et al., 2019), and *Paraglaciecola hydrolytica* S66 (50°C; Visnapuu et al., 2020). As for the thermostability profile (Figure 3B), the activity dropped rapidly after incubation at temperatures above 40°C, suggesting the poor thermostability of NAGaseA, which was similar to that of GH20 GlcNAGases from *C. meiyuanensis* (Zhang et al., 2020b), *Microbacterium* sp. HJ5 (Zhou et al., 2017b) and *Aeromonas* sp. 10S-24 (Ueda et al., 2000).

Regarding the effect of pH, NAGaseA exhibited the optimum pH of 6.5 (Figure 3C). The optimal pH value of NAGaseA was higher than some reported NAGases, such as NAGase from *C. meiyuanensis* (5.4; Zhang et al., 2020b), *Salmonella enterica* (4.0; Herlihey et al., 2014) and *Lactobacillus casei* (5.0; Tews et al., 1996a). In addition, NAGaseA retained excellent activity after incubation 120 h under the corresponding buffers of pH 6–10, indicating that NAGaseA possessed good pH stability compared to other reported NAGases (Figure 3D; Morimoto et al., 2002; Lan et al., 2004; Chen et al., 2015).

### Effects of Metal Ions on Activity of Recombinant NAGaseA

Many reports have shown that metal ions affected enzymatic activity. Thus, the effects of various metal ions on NAGaseA activity were also investigated. As shown in Table 1, the enzyme retained approximately 96% of its initial activity after incubation in 10 mM EDTA, suggesting that EDTA did not inhibit the enzymatic activity and NAGaseA is non-metal dependent. Cu^2+^ showed a great inhibition effect on the activity of NAGaseA, which was similar to that of NAGases from *A. caviae* (Cardozo et al., 2017) and *C. meiyuanensis* (Zhang et al., 2018). Besides, NAGaseA activity was partially inhibited by Fe^3+^ and Co^2+^, NAGases from *R. miehei* and *Streptomyces alfalfa* shared the same profile as reported (Yang et al., 2014; Lv et al., 2019).

**TABLE 1:** Effects of metal ions on the activity of NAGaseA.

Metal ions	Chemicals	Concentration (mM)	Relative activity (%)
No addition	–	0	100
Cu^2+^	CuCl_2_	10	24.27 ± 2.42
Fe^3+^	FeCl_3_	10	45.19 ± 2.25
Co^2+^	CoCl_2_	10	78.55 ± 5.49
Ni^2+^	NiCl_2_	10	92.95 ± 7.43
Ca^2+^	CaCl_2_	10	94.01 ± 1.88
Al^3+^	AlCl_3_⋅6H_2_O	10	91.97 ± 3.67
Mg^2+^	MgCl_2_	10	90.19 ± 6.31
Zn^2+^	ZnCl_2_	10	93.07 ± 4.65
Mn^2+^	MnCl_2_	10	98.98 ± 2.96
EDTA	EDTA	10	95.71 ± 4.78

### Substrate Specificity of NAGaseA

The substrate specificity of NAGaseA was measured using standard assay conditions. As depicted in Table 2, NAGaseA exhibited the highest specific activity toward *p*NP-GlcNAc, with a specific activity of 333.33 U/mg. Among (GlcNAc)_2–6_, NAGaseA showed the highest activity toward (GlcNAc)_2_, followed by (GlcNAc)_3_, (GlcNAc)_4_, (GlcNAc)_5_, and (GlcNAc)_6_, which showed that the specific activity toward *N*-acetyl COSs decreased with increasing degree of polymerization (Ogawa et al., 2006). Besides, little activity (0.0037 U/mg) was detected using colloid chitin as substrate, which was agreed with other reported GH20 NAGases that exhibited little hydrolysis activity toward chitin substrate without the cooperation with other chitinases (Zhou et al., 2017b). Moreover, no activity was observed when chitosan, chitin power, CMC was used as the substrates. These results indicated that NAGaseA possessed the typical NAGase activity with strict substrate specificity.

**TABLE 2:** Substrate specificity of NAGaseA.

Substrates	Specific activity (U/mg of protein)
Colloidal chitin	0.0037 ± 0.00047
Chitosan	N.D.
Chitin power	N.D.
*p*NP-GlcNAc	333.33 ± 19.21
(GlcNAc)_2_	201.68 ± 11.69
(GlcNAc)_3_	152.84 ± 7.18
(GlcNAc)_4_	81.34 ± 5.49
(GlcNAc)_5_	55.52 ± 2.11
(GlcNAc)_6_	23.59 ± 1.13

In addition, the kinetic parameters for NAGaseA were also measured with *pNP*-GlcNAc as the substrate. The results showed that the *V*_max_, *K*_m_, *k*_cat_, and *k*_cat_/*K*_m_ for NAGaseA were 3333.33 μmol min^–1^ l^–1^, 39.99 μmol l^–1^, 4667.07 s^–1^, and 116.71 ml μmol ^–1^ s^–1^, respectively.

### Hydrolysis Mechanism of NAGaseA Toward Colloid Chitin and *N*-Acetyl COSs

**FIGURE 4:**
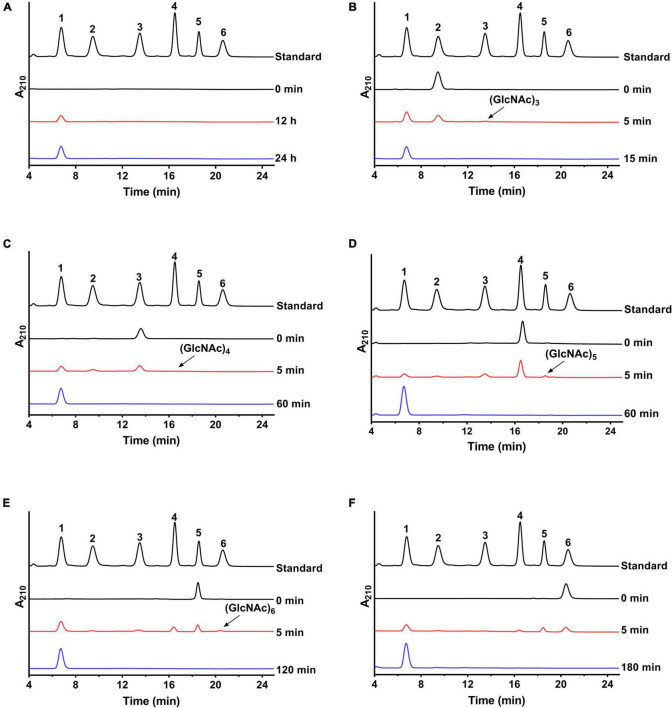
Hydrolysis analysis of NAGaseA toward colloid chitin and *N*-Acetyl COSs. **(A)** Colloid chitin and **(B–F)** (GlcNAc)_2–6_ were performed in PBS (pH 6.5) at 40°C contained 60 ng NAGaseA and 10 g/l various substrate. Numbers 1–6 represent GlcNAc to (GlcNAc)_6_.

The hydrolysis patterns of colloid chitin and *N*-acetyl COSs by NAGaseA were measured (Figure 4). As shown in Figure 4A, GlcNAc was the sole product hydrolyzed by colloid chitin, with its concentration raised as hydrolysis time increased. In the hydrolysis process of NAGaseA, (GlcNAc)_2_ was converted to GlcNAc as the sole product (Figure 4B) (GlcNAc)_3_ to (GlcNAc)_2_ and GlcNAc (Figure 4C), (GlcNAc)_4_ to (GlcNAc)_3_, (GlcNAc)_2_ and GlcNAc (Figure 4D), (GlcNAc)_5_ to (GlcNAc)_4_, (GlcNAc)_3_, (GlcNAc)_2_ and GlcNAc (Figure 4E), and (GlcNAc)_6_ was converted to (GlcNAc)_5_, (GlcNAc)_4_, (GlcNAc)_3_, (GlcNAc)_2_ and GlcNAc (Figure 4F) at the initial incubation within 5 min. Furthermore, NAGaseA could hydrolyze (GlcNAc)_2_-(GlcNAc)_6_ into pure GlcNAc after an incubation time of 15–180 min, respectively. The overall rates of hydrolysis were in the order: (GlcNAc)_2_ > (GlcNAc)_3_ > (GlcNAc)_4_ > (GlcNAc)_5_ > (GlcNAc)_6_, which was in accordance with the results of substrate specificity. These results indicated that NAGaseA is a typical exo-NAGase.

In addition, minor (GlcNAc)_3_, (GlcNAc)_4_, (GlcNAc)_5_, and (GlcNAc)_6_ were also produced from (GlcNAc)_2_, (GlcNAc)_3_, (GlcNAc)_4_, and (GlcNAc)_5_ in short reaction times. These results indicated that NAGaseA is capable of producing higher *N*-acetyl COSs ((GlcNAc)_3_-(GlcNAc)_6_) from (GlcNAc)_2_-(GlcNAc)_5_, which exhibited transglycosylation activity. Our previous study also reported that *Cm*NAGase from *Chitinolyticbacter meiyuanensis* SYBC-H1 could produce higher *N*-acetyl COSs (GlcNAc)_3_-(GlcNAc)_7_ from (GlcNAc)_2_-(GlcNAc)_6_, respectively (Zhang et al., 2020b). However, unlike *Cm*NAGase, no new peak was presumed as (GlcNAc)_7_ generated when using (GlcNAc)_6_ as the substrate, which could be attributed to the lower reverse hydrolysis activity of NAGaseA.

### Synergistic Action Between NAGaseA and Chitinases on Chitin Degradation

**FIGURE 5:**
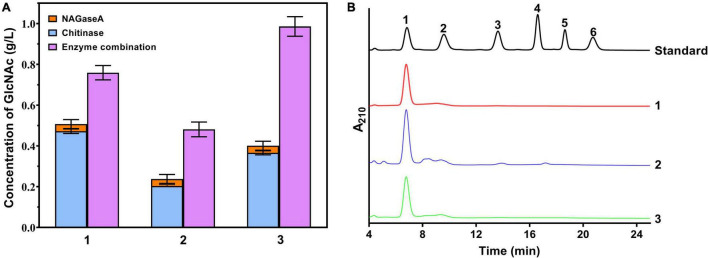
Production GlcNAc from chitin using cocktail enzyme. **(A)** Effect of cooperative interaction between NAGaseA with chitinases on the hydrolysis of chitin. 1: cooperation of NAGaseA with purified chitinase chiA; 1: cooperation of NAGaseA with purified chitinase chiA; 2: cooperation of NAGaseA with crude enzyme from *C. meiyuanensis* SYBC-H1; and 3: cooperation of NAGaseA with crude enzyme from *Chitinibacter sp*. GC72. **(B)** HPLC analysis of the finial products of reaction mixture.

To investigate the potential application of NAGaseA in GlcNAc production, the synergistic action between NAGaseA and other chitinases on chitin degradation was studied. As illustrated in Figure 5, the released reducing sugar concentrations from the cooperation of NAGaseA with purified chitinase chiA, the crude enzyme from *C. meiyuanensis* SYBC-H1, and crude enzyme from *Chitinibacter sp*. GC72 were 0.759, 0.481, and 0.986 g/l, which were 1.61-, 2.36-, and 2.69-fold that of the concentration of the two enzymes accumulated, respectively. Among, NAGaseA behaved the best to improve efficiency with the crude enzyme from GC72, which could be attributed to the better synergistic effect with other chitinases from *Chitinibacter* sp. GC72. Zhou et al. reported a combination of commercial chitinase CtnSg and NAGase rHJ5Nag used for chitin degradation, with an improvement rate of 2.02-fold (Zhou et al., 2017b). Chenyin Lv et al. also investigated the synergistic action between commercial chitinase SgCtn and NAGase SaHEX, which obtained higher production of reducing sugars than the single enzyme for SgCtn (4.3-fold) and SaHEX (8.1-fold; Lv et al., 2019). In our study, NAGaseA can not only combine with purified chitinase but also crude chitinases in the production of GlcNAc from chitin. Moreover, it was worth noting that GlcNAc purity of 96% was obtained and few other *N*-acetyl COSs were detected in the final reaction mixture. Li et al. (2021) obtained the GlcNAc with a purity of 99.7% using colloidal chitin as the substrate under the co-action of two chitinases after 24 h of incubation, Du et al. (2019) reported the maximum GlcNAc yields of 87% using two enzyme combination during 2.25 h. These results indicate that NAGaseA has great potential in the production of GlcNAc in the multienzyme combination system.

## Conclusion

In this study, the gene encoding a GH20 family β-*N*-acetylglucosaminidase NAGaseA from the chitinolytic bacterium *Chitinibacter* sp. GC72 was cloned and functionally expressed. The domain structure prediction showed that NAGaseA contains GH20 family catalytic domain and exhibited low similarity with reported GH20 NAGases. Analysis from the HPLC revealed that NAGaseA was a multifunctional NAGase exhibited the exo-acting activity and trans-glycosylation activity. Furthermore, NAGaseA also behaved with excellent synergistic performance with other chitinases during the degradation of colloidal chitin, and high purity of GlcNAc was obtained as the final product. These results indicated that NAGaseA has great potential in the bioconversion of chitin waste and behaved as an excellent candidate in GlcNAc production.

## Data Availability Statement

The original contributions presented in the study are included in the article/Supplementary Material, further inquiries can be directed to the corresponding author.

## Author Contributions

YC and NZ designed the experiments, carried out the experiments, and drafted the manuscript. XC and GW participated in experiments. AZ conceived the idea and revised the manuscript. KC and PO proofread the manuscript. All authors read and approved the finally manuscript.

## Conflict of Interest

The authors declare that the research was conducted in the absence of any commercial or financial relationships that could be construed as a potential conflict of interest.

## Publisher’s Note

All claims expressed in this article are solely those of the authors and do not necessarily represent those of their affiliated organizations, or those of the publisher, the editors and the reviewers. Any product that may be evaluated in this article, or claim that may be made by its manufacturer, is not guaranteed or endorsed by the publisher.

## Footnotes

1
http://www.snapgene.com/

2
http://web.expasy.org/protparam/

3
http://blast.ncbi.nlm.nih.gov/Blast.cgi

4
http://prosite.expasy.org/scanprosite/

5
http://www.cbs.dtu.dk/services/SignalP/

6
http://espript.ibcp.fr/ESPript/cgi-bin/ESPript.cgi

7
http://raptorx.uchicago.edu/StructPredV2/predict/
