# Supplementary material for: Characterization of a New Multifunctional GH20 β-N-Acetylglucosaminidase From Chitinibacter sp. GC72 and Its Application in Converting Chitin Into N-Acetyl Glucosamine
Source: Front Microbiol. 2022 May 10;13:874908. doi: 10.3389/fmicb.2022.874908 (PMC9129912; doi:10.3389/fmicb.2022.874908)
Supplement: Supplementary file 1 [file Table_1.DOCX]

**Characterization of a new multifunctional GH20** **β-*N*-acetylglucosaminidase from *Chitinibacter sp*. GC72 and its** **application in converting chitin into *N-*acetyl glucosamine**

Yan Chen^a,†^, Ning Zhou^a,†^, Xueman Chen^a^, Guoguang Wei^a^, Alei Zhang^a,b,^*, Kequan Chen^a^ , Pingkai Ouyang^a^

*^a^State Key Laboratory of Materials-Oriented Chemical Engineering, College of Biotechnology and Pharmaceutical Engineering, Nanjing Tech University, Nanjing 211816, China.*

*^b^Jiangsu Key Laboratory of Marine Bioresources and Environment, Jiangsu Ocean University, Lianyungang, 222005, China.*

*Corresponding author.

1. mail address: zhangalei@njtech.edu.cn

^†^These authors have contributed equally to this work and share first authorship

**Table S1.** Purification of recombinant NGAseA.

| Purification method | Total activity  (U) | Total protein  (mg) | Specific activity (U/mg) | Purification (fold) | Recovery yield (%) |
| --- | --- | --- | --- | --- | --- |
| Crude enzyme | 16048.18 | 59.4 | 270.17 | 0 | 100 |
| Ni–NTA resin | 12613.87 | 33.7 | 373.29 | 1.39 | 78.6 |

**Table S2.** Half-lives of recombinant *NAGaseA*.

| Temperature (ºC) | Half-life (h) |
| --- | --- |
| 30 | 13.9±0.65 |
| 37 | 7.9±0.24 |
| 40 | 7.2±0.33 |
| 45 | 0.58±0.05 |


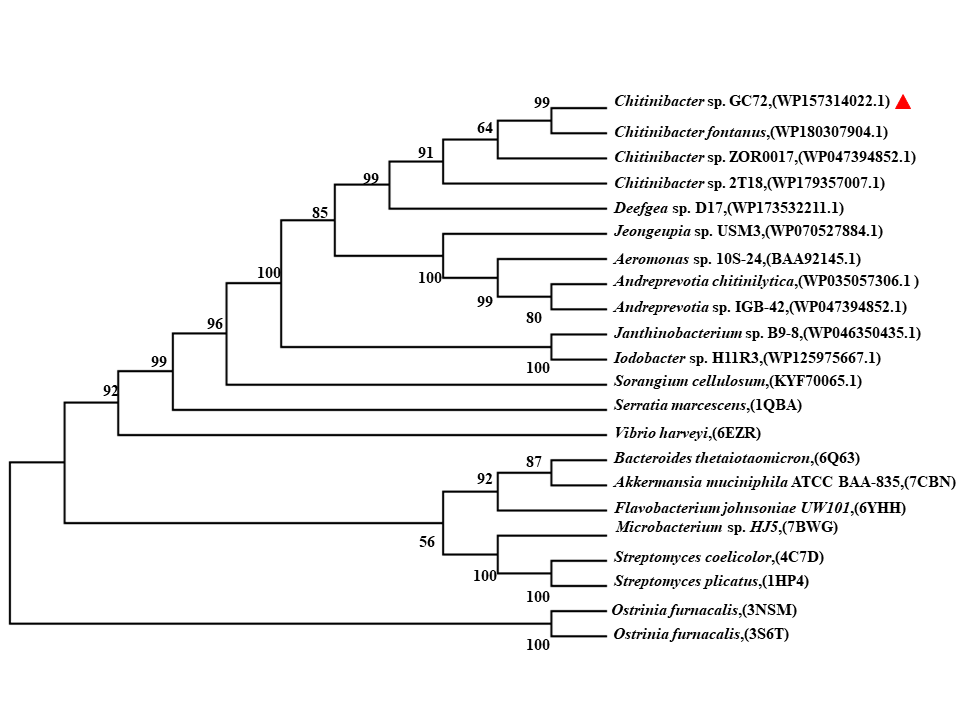


**Figure S1.** Phylogenetic relationships between NAGaseA and other bacterial GH20 NAGases. The phylogenetic tree was constructed by the neighbor-joining algorithm based on the amino acid sequence alignment in MEGA 7.0.


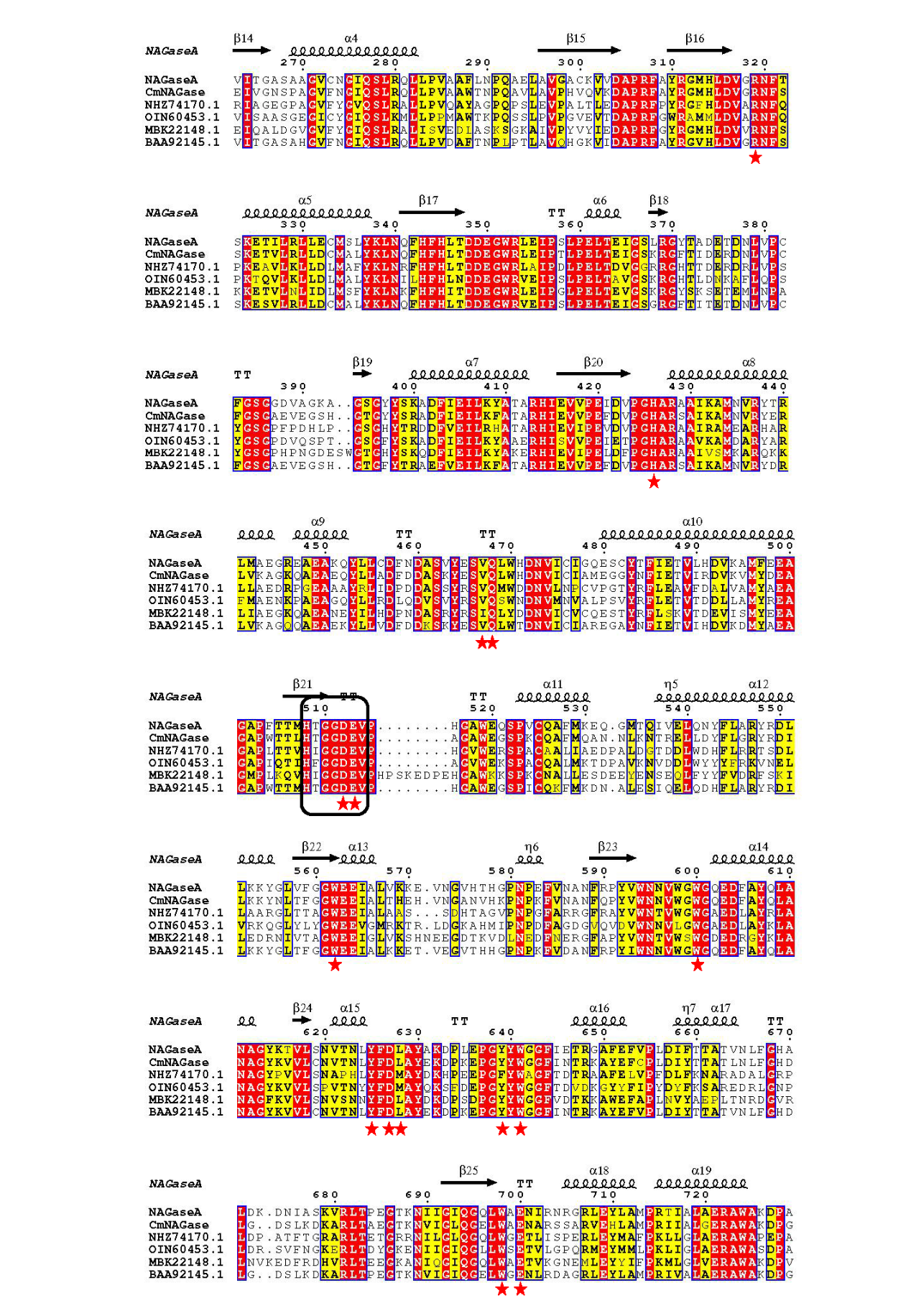


**Figure S2.** Partial amino acid sequences and structures alignments of NAGaseA with other GH20 NAGases. Similar sequences are marked by pentagrams and identical sequences are highlighted in red and yellow. Sequences are shown as follows: BAA92145: NAGase from *Aeromonas* sp. 10S-24 (accession no. BAA92145); *Cm*NAGase: the GlcNAcase from *Streptomyces coelicolor* A3(2); NHZ74170: the NAGase from *Microbacterium sp.* HJ5; OIN60453: the NAGase from *Vibrio harveyi*; MBK22148: the NAGase from *Serratia marcescens.*
